# The present and future research agenda of sporotrichosis on the silver anniversary of zoonotic sporotrichosis in Rio de Janeiro, Brazil

**DOI:** 10.1590/0074-02760230208

**Published:** 2024-02-12

**Authors:** Rodrigo Almeida-Paes, Antonio Carlos Francesconi do Valle, Dayvison Francis Saraiva Freitas, Priscila Marques de Macedo, Rosely Maria Zancopé-Oliveira, Maria Clara Gutierrez-Galhardo

**Affiliations:** 1 Fundação Oswaldo Cruz-Fiocruz, Instituto Nacional de Infectologia Evandro Chagas, Laboratório de Micologia, Rio de Janeiro, RJ, Brasil; 2 Fundação Oswaldo Cruz-Fiocruz, Instituto Nacional de Infectologia Evandro Chagas, Laboratório de Dermatologia Infecciosa, Rio de Janeiro, RJ, Brasil

**Keywords:** Sporothrix brasiliensis, one health, public health, research

## Abstract

Twenty-five years have passed since the initial observation of endemic zoonotic sporotrichosis in Rio de Janeiro, Brazil. Since then, this disease has spread throughout South America. Accompanying the emergence of this mycosis, some progress has been made, including the expansion of a research network in this field and higher visibility of sporotrichosis within government authorities and funding agencies. However, there are still some challenges to curbing the expansion of this disease in the coming years. These include the development of rapid and accurate diagnostic tests, new antifungal drugs, particularly for the treatment of extracutaneous manifestations of sporotrichosis, and more comprehensive care for cats with sporotrichosis. Including these actions in the sporotrichosis research agenda is required so as to change the development of this disease in the years to come.

Sporotrichosis is a subacute or chronic mycosis of animals and humans caused by some species of the genus *Sporothrix*. This genus belongs to the family Ophiostomataceae in the order Ophiostomatales. *Sporothrix* spp. are saprobic fungi, meaning that they obtain their nutrients by breaking down dead organic matter present in soil, decaying plants, and moss, among other sources. There are over 50 species in the genus *Sporothrix*, but only a few are known to cause disease in humans. The most common species of *Sporothrix* that cause sporotrichosis in humans are *Sporothrix schenckii*, *Sporothrix brasiliensis*, *Sporothrix globosa*, *Sporothrix mexicana*, and *Sporothrix luriei*. Sporotrichosis is usually transmitted to humans through contact with contaminated soil and plant material. The fungus can enter the body through a break in the skin, such as a cut, scratch, or puncture wound. Moreover, sporotrichosis also manifests as a zoonotic disease, being transmitted in some areas through the bite or scratch of an infected cat.^1^

Sporotrichosis, also known as the “rose gardener’s disease”, is globally distributed, but it is most frequent in tropical and subtropical regions. The disease is particularly common in Latin America, but also usual in Africa, Asia, and Australia.^2^ In recent years, there has been a significant increase in the number of sporotrichosis cases reported in many countries.^3^ This increase has been attributed to a number of factors, including heightened awareness of the disease among healthcare providers; changes in environmental conditions that favour the growth and survival of *Sporothrix* spp.; increased urbanisation and deforestation that have brought humans into closer contact with *Sporothrix*-containing soil and plants; and the escalating prevalence of domestic cats falling sick and subsequently transmitting the disease.

The increasing number of publications on sporotrichosis reflects the growing interest in this field, particularly in clinical and applied research. It is interesting to note that a recent work pointed out that most studies in the sporotrichosis field, from 1945 to 2018,^3^ had been published after 2000. In the year 2000 Memórias do Instituto Oswaldo Cruz published a paper describing outbreaks of cat-transmitted sporotrichosis in Rio de Janeiro, Brazil, which had started in 1998.^4^ Since then, Brazil has become the leading country in sporotrichosis research collaboration and networking, with co-authorships in 45 countries.^3^

Twenty-five years after the emergence of zoonotic sporotrichosis, we aim to describe its profile and the progress made in its management, as well as the challenges that remain to reduce the burden of this disease and to improve the quality of life for humans and animals infected with *Sporothrix* spp.

*The beginning of the story* - The first publication by our group, “Sporotrichosis: an emergent soonosis in Rio de Janeiro”, described an unprecedented epidemic of sporotrichosis involving 66 humans, 117 cats, and seven dogs registered at our institution, previously known as Hospital Evandro Chagas and currently known as Instituto Nacional de Infectologia Evandro Chagas (INI), Fundação Oswaldo Cruz (Fiocruz), from July 1998 to July 2000.^4^ Most human cases (78.8%) reported contact with sick cats, including scratching or biting in 47% of cases. A pivotal case in noticing this new epidemiologic scenario of sporotrichosis was that of a veterinarian who developed the infection after a scratch from a cat with wounds. This incident was also brought to the attention of the newly established Zoonosis Service at INI, which had been created in the late 1990s to address emerging zoonotic threats. The cat, which was also diagnosed with sporotrichosis, was adopted by the veterinarian responsible for the service and successfully treated. This case highlighted the potential for zoonotic transmission of sporotrichosis from cats and underscored the need for specialised services to address this emerging public health concern. These data contrasted with the 13 cases diagnosed from 1987 to 1998. However, we have stated that the zoonotic sporotrichosis epidemic may have begun earlier, as two of these cases (one in 1991 and another in 1997) reported that their lesions appeared at a site previously scratched by a sick cat. Other institutions in Rio de Janeiro also reported similar cases before 1998.^1^

The number of human and cat cases continued to increase in subsequent years, and INI/Fiocruz became the major reference centre for sporotrichosis in Rio de Janeiro. By georeferencing our human cases up to 2007 and analysing their socio-spatial distribution, we identified a “belt” of cases along the boundaries between the capital and its periphery (“Baixada Fluminense”). These areas were characterised by deforestation, urbanisation, high population density, precarious sanitation, and poverty. We emphasised at this point that a neglected urban epidemic had been established and that there were no specific measures aimed at controlling it.^5^ A later study in the municipality of Duque de Caxias found that the endemic area of sporotrichosis was associated with low *per capita* income and a lack of domestic supply of treated water.^6^

In 2013, sporotrichosis became a notifiable disease in the State of Rio de Janeiro.^7^ One of the public health actions taken by the government was the decentralisation of clinical support for patients with sporotrichosis, who began to receive medical care at primary centres, with only the most severe cases referred to our tertiary outpatient clinics at INI-Fiocruz. In addition, infrastructure for veterinary care of cat cases was established.

From 2011 to April 28, 2023, 14,490 human cases and 20,202 cat cases of sporotrichosis were reported to the Notifiable Diseases Information System (Sinan) of the Rio de Janeiro State Health Department (SES/RJ) in almost all municipalities of the state. At our institution, 5,456 human cases were attended from 1998 to July 2023.^8^ Currently, sporotrichosis fits the classification criteria, among other deep mycoses, of a neglected tropical disease (NTD) by the World Health Organization (WHO).^9^ Unfortunately, no *Sporothrix* species is included in the WHO fungal priority pathogens list to guide research, development, and public health action.^10^

*Current aspects of zoonotic sporotrichosis in RJ* - The increasing number of sporotrichosis cases in Rio de Janeiro during the first decade of zoonotic sporotrichosis in our state, led to the discovery that this mycosis could be caused by species other than *S. schenckii*. In fact, the study that described these other human pathogenic *Sporothrix* species included 29 strains from the epidemic, which clustered together in a clade further named *S. brasiliensis*.^11^ This emergent species is most commonly found in Brazil, where it was first identified, but it has since been reported in other countries in South America,^12^ as well as in Europe.^13^ The expansion and knowledge of the distinct geographic distribution of *S. brasiliensis* is thought to be due to a number of factors, including climate change, deforestation, and the urbanisation of rural areas.

*Sporothrix brasiliensis* is considered a highly virulent species, meaning that it is more likely to cause severe disease than other *Sporothrix* species. For example, experimental animal models of *S. brasiliensis* infection show higher mortality, tissue damage, and fungal burden than infection caused by other species.^14^ This reflects in the clinical manifestations of human sporotrichosis due to *S. brasiliensis*. In fact, several uncommon clinical aspects of sporotrichosis, many of them severe, have been described during the last 25 years of endemic zoonotic sporotrichosis in Rio de Janeiro.^15^ ^,^ ^16^ ^,^ ^17^ ^,^ ^18^ ^,^ ^19^

Most transmission of zoonotic sporotrichosis in Rio de Janeiro occurs within the patient’s home, and housewives, older people, and children are the most affected due to closer contact with sick cats.^1^ The classic clinical forms of sporotrichosis (lymphocutaneous and fixed cutaneous forms) are the most common.^20^ However, disseminated cutaneous forms (without immunosuppression), external ocular sporotrichosis, and hypersensitivity manifestations (reactive arthritis, erythema nodosum, erythema multiforme, and Sweet’s syndrome) are unique to this form of transmission associated with *S. brasiliensis*.^16^ ^,^ ^21^ Multifocal sporotrichosis with bone, mucosal, and central nervous system involvement has mostly been observed in immunosuppressed patients.^15^ ^,^ ^17^ ^,^ ^18^ Notably, during this period, sporotrichosis has emerged as an opportunistic infection in people living with human immunodeficiency virus / acquired immunodeficiency syndrome (HIV/AIDS), as their endemic areas now overlap.^22^ Meningeal involvement in this population is associated with poor prognosis and a higher mortality risk.^17^ Such multifaceted manifestations are seldom reported with other sporotrichosis agents.

*Advances in the sporotrichosis field in the last 25 years* - The most severe cases of *S. brasiliensis* infection require multidisciplinary care involving several medical specialists.^20^ In Rio de Janeiro, this level of care is available at university hospitals and at our referral centre. These forms require hospitalisation, and many of them progress to death.^23^ Immunosuppressed patients are faced with situations that were unthinkable before this unprecedented zoonosis.^22^ The involvement of several different areas in the management of sporotrichosis cases has led to an increase in the quantity and quality of human resources in medical, veterinary and research fields.

The unprecedented scenario of zoonotic sporotrichosis has undoubtedly raised the visibility of the disease among clinicians and researchers, but also among people involved in the development of public health policies. As mentioned before, our state government has taken actions to facilitate access to adequate medical care and treatment for patients. This model, which was replicated at least in part by other government authorities in Brazil, included the following: making human sporotrichosis a regional notifiable disease; managing regular cases in primary care centres; referring patients with unusual forms to tertiary centres, such as those related to immunosuppression, pregnancy, and hypersensitivity forms; and providing free distribution of itraconazole for human treatment. Although these measures are still not enough to control the expansion of cases, they do represent an advance in combating the disease. Some Brazilian cities also conduct active surveillance for sporotrichosis cases.^24^ This has not yet been possible in Rio de Janeiro due to the high number of cases.

Another achievement in this field was the inclusion of sporotrichosis on the agenda of several funding agencies. In fact, an analysis from 2012 to 2018 showed that 457 out of 1,310 publications on *Sporothrix* declared receiving funding.^3^ Five of the most acknowledged funding agencies were from Brazil, two from Mexico, one from China, one from the United States, and one from South Africa, demonstrating that this engagement was not restricted to hyperendemic areas of zoonotic sporotrichosis.

*Challenges to face in the coming years* - Although classical sporotrichosis forms can easily be diagnosed from the culture of clinical samples or even by the clinical and epidemiological characteristics of the patients, alternative diagnostic methods are required for fast and reliable diagnosis of disseminated and extracutaneous sporotrichosis.^1^ Several methods for presumptive sporotrichosis diagnosis by antibody detection have been developed in the past 25 years, but they still have sensitivity limitations and are not approved for sale by the Brazilian regulatory agency (ANVISA).^25^ These tests, most of which are in an enzyme-linked immunosorbent assay (ELISA) format, require some laboratory facilities and take some time to yield results. Therefore, there is still a need for faster and more effective diagnostic methods, especially in life-threatening situations such as meningitis caused by *Sporothrix* spp.^26^

Both immunologic and molecular tests have been developed for these cases, but they still lack validation and wide distribution across laboratories. For instance, a lateral flow assay has been developed to detect anti-*Sporothrix* antibodies in humans. Although it was tested with patients with zoonotic sporotrichosis,^27^ it is likely that this test will also diagnose humans infected in the environment or by inhalation of conidia, as well as patients infected with *S. schenckii* or *S. globosa*. Quantitative polymerase chain reaction (qPCR) aiming to detect *Sporothrix* mitochondrial DNA or even to differentiate sporotrichosis agents were also developed.^28^ ^,^ ^29^ We believe that the commercialisation of those tests after regulatory approvals will improve the diagnosis of this disease, since those methods do not require specialised mycological training by laboratory professionals.

Additionally, antigen detection is a highly effective diagnostic tool for several mycoses, providing quick and reliable diagnosis for cryptococcosis and histoplasmosis, for instance.^25^ However, as *per* our current knowledge, there are still no antigen detection tests for sporotrichosis, even within research centres. The pathogenic *Sporothrix* species exhibit rhamnomannan in their cell walls,^30^ a distinctive molecular feature not shared with other clinically relevant pathogenic fungi. This unique characteristic makes it a promising target for future research endeavours in the development of diagnostic tests for sporotrichosis.

Another important current limitation for sporotrichosis is treatment. Although cutaneous sporotrichosis is effectively treated with oral antifungals such as itraconazole, terbinafine and saturated solution of potassium iodide,^1^ managing disseminated forms can be challenging. Amphotericin B is the only intravenous antifungal drug described in the available guidelines to treat severe sporotrichosis. However, 20% of patients must discontinue treatment due to adverse reactions, and the cure rate for severe forms is around 50%.^31^ Cerebrospinal fluid sterilisation is arduous to achieve with intravenous amphotericin B alone, and relapses are common.^17^ Ongoing research endeavours are exploring solutions to this challenge, unveiling the anti-*Sporothrix* potential of novel antifungal drugs like isavuconazole and olorofim.^32^ ^,^ ^33^ Isavuconazole has already been successfully used for treating various mycoses, including the challenging mucormycosis, while olorofim is still awaiting approval from some regulatory agencies. Promising candidates for repurposing, such as pentamidine, and newly synthesised drugs like quinone derivatives, are also under scrutiny.^34^ ^,^ ^35^ It is imperative to bridge the gap between this evolving knowledge and clinical practice by initiating clinical trials. These trials would evaluate the efficacy of these new drugs, especially in patients with severe forms of sporotrichosis, translating research findings into tangible advancements in patient care.

The most challenging issue to be faced in reducing the sporotrichosis burden is the fate of infected cats. Of the 2,926 cases of human sporotrichosis in our institution in which the outcome of the sick cat was reported in the medical records, only 568 (19.4%) were managed appropriately: either the animal was being treated by its owners; or surrendered to an animal organisation for treatment; or euthanised and cremated. The rest were simply abandoned or died without receiving proper cremation. In Brazil, animal abandonment has been a crime since 1998, when Federal law number 9.605/98 was published. The penalty for this crime was increased by Federal law 14.064/20. Animal abandonment promotes the sustained spread of this mycosis, with the fungus remaining in the environment, where it can infect other cats or even humans and dogs.

It is also crucial to rectify the misperception regarding the role of cats in the dynamics of sporotrichosis in Brazil and communicate it appropriately, not only to health professionals, managers, and researchers, but also to the population. Contrary to the belief that *S. brasiliensis* cases originated and were exported solely from Rio de Janeiro, evidence indicates that the emergence of this disease was predated by the presence of the fungus in various areas. For instance, *S. brasiliensis* was identified in soil samples collected in Argentina,^36^ highlighting its presence in locations distinct from Rio de Janeiro. Moreover, the circulating *S. brasiliensis* genotype in Brasilia, situated approximately 1,000 km from Rio de Janeiro, diverged from a common ancestor around 2.2 million years ago.^37^ This timeline suggests that the dissemination of this highly pathogenic *Sporothrix* species was not solely facilitated by cats. Two of our cases before 1998, predating the epidemic, had a history of feline transmission, occurring seven years and a year prior. It is plausible that during that period, conducive conditions for the transmission of a virulent species to a susceptible host, such as the cat, were being established in our city. The establishment of those conditions may now be beginning in other places. In addition, there are descriptions of sporotrichosis due to *S. brasiliensis* in patients infected with environmental sources that deny any contact with cats.^38^

Public health measures were implemented only 16 years after the outbreak’s onset, when it had already reached hyperendemic levels. This delay is a recurring pattern for neglected diseases. However, the severity and high number of cases in Rio de Janeiro prompted better recognition and diagnosis of similar cases in other states, leading to some timely public health interventions that partially curbed the disease’s spread.^39^ Making sporotrichosis a notifiable disease nationwide may bring more realistic visibility of the current situation, helping to provide resources and efforts for its control. Molecular and epidemiological studies on animal and environmental samples, in the one health context, are necessary to convince people that, akin to humans, cats are victims of *S. brasiliensis*.

*In conclusion* - On this silver anniversary of zoonotic sporotrichosis in Rio de Janeiro, Brazil, there are some accomplishments to celebrate, but other challenges to address in order to change the situation of this disease before its gold anniversary. It is also important to highlight that sporotrichosis is no longer a disease of universities and research centres, but an important public health problem which needs to be faced by all sectors of public health systems.
